# High-Speed Real-Time Resting-State fMRI Using Multi-Slab Echo-Volumar Imaging

**DOI:** 10.3389/fnhum.2013.00479

**Published:** 2013-08-26

**Authors:** Stefan Posse, Elena Ackley, Radu Mutihac, Tongsheng Zhang, Ruslan Hummatov, Massoud Akhtari, Muhammad Chohan, Bruce Fisch, Howard Yonas

**Affiliations:** ^1^Department of Neurology, School of Medicine, The University of New Mexico, Albuquerque, NM, USA; ^2^Department of Electrical and Computer Engineering, The University of New Mexico, Albuquerque, NM, USA; ^3^Department of Physics and Astronomy, The University of New Mexico, Albuquerque, NM, USA; ^4^Department of Physics, University of Bucharest, Bucharest, Romania; ^5^Division of Psychiatry and Neuroscience, Walter Reed Army Institute of Research, Silver Spring, MD, USA; ^6^Semel Institute for Neuroscience and Human Behavior, University of California Los Angeles, Los Angeles, CA, USA; ^7^Department of Neurosurgery, School of Medicine, The University of New Mexico, Albuquerque, NM, USA

**Keywords:** real-time resting state fMRI, multi-slab echo-volumar imaging, independent component analysis (ICA), seed-based functional connectivity, cerebrovascular pulsatility, epilepsy, brain tumor, arteriovenous malformation

## Abstract

We recently demonstrated that ultra-high-speed real-time fMRI using multi-slab echo-volumar imaging (MEVI) significantly increases sensitivity for mapping task-related activation and resting-state networks (RSNs) compared to echo-planar imaging (Posse et al., 2012). In the present study we characterize the sensitivity of MEVI for mapping RSN connectivity dynamics, comparing independent component analysis (ICA) and a novel seed-based connectivity analysis (SBCA) that combines sliding-window correlation analysis with meta-statistics. This SBCA approach is shown to minimize the effects of confounds, such as movement, and CSF and white matter signal changes, and enables real-time monitoring of RSN dynamics at time scales of tens of seconds. We demonstrate highly sensitive mapping of eloquent cortex in the vicinity of brain tumors and arterio-venous malformations, and detection of abnormal resting-state connectivity in epilepsy. In patients with motor impairment, resting-state fMRI provided focal localization of sensorimotor cortex compared with more diffuse activation in task-based fMRI. The fast acquisition speed of MEVI enabled segregation of cardiac-related signal pulsation using ICA, which revealed distinct regional differences in pulsation amplitude and waveform, elevated signal pulsation in patients with arterio-venous malformations and a trend toward reduced pulsatility in gray matter of patients compared with healthy controls. Mapping cardiac pulsation in cortical gray matter may carry important functional information that distinguishes healthy from diseased tissue vasculature. This novel fMRI methodology is particularly promising for mapping eloquent cortex in patients with neurological disease, having variable degree of cooperation in task-based fMRI. In conclusion, ultra-high-real-time speed fMRI enhances the sensitivity of mapping the dynamics of resting-state connectivity and cerebro-vascular pulsatility for clinical and neuroscience research applications.

## Introduction

Mapping of intrinsic signal variation mostly in the low-frequency band<0.1 Hz has emerged as a powerful tool and adjunct to task-related fMRI and fiber tracking based in diffusion tensor imaging (DTI) for mapping functional connectivity within and between resting-state networks (RSNs) (Fox et al., 2005; De Luca et al., 2006; Raichle and Snyder, 2007; Schopf et al., 2010; Li et al., 2011). Recent studies have shown that dozens of different RSNs can be measured across groups of subjects (Abou-Elseoud et al., 2010; Allen et al., 2011). Anti-correlations between the default mode network (DMN) and task-positive networks provide insights into competitive mechanisms that control resting-state fluctuations (Fox et al., 2005; Uddin et al., 2009). There is increasing evidence that RSNs are not stationary (Hou et al., 2006; Kang et al., 2011) and that correlations with fluctuations in other measurements, such as α-power in EEG (Wu et al., 2010) and transient (∼100 ms) topographies of EEG current source densities (microstates) (Britz et al., 2010; Laufs, 2010; Lehmann, 2010; Musso et al., 2010; Van de Ville et al., 2010) exist. Variations in ongoing activity have been shown to predict changes in task performance and alertness, highlighting their importance for understanding the connection between brain activity and behavior (Eichele et al., 2008; Sadaghiani et al., 2010). Resting-state correlation mapping has been shown to be a promising tool for reliable functional localization of eloquent cortex in healthy controls, and patients with brain tumors and epilepsy (Liu et al., 2009; Zhang et al., 2009; Mannfolk et al., 2011; Stufflebeam et al., 2011). It has been suggested that this task-free paradigm may provide a powerful approach to map functional anatomy in patients without task compliance, which allows multiple brain systems to be determined in a single scanning session (Liu et al., 2009). Recent studies have investigated non-stationarity, which is prominent in the resting state, and demonstrated dynamic changes in network connectivity (Chang and Glover, 2010; Sakoglu et al., 2010; Kiviniemi et al., 2011). There is now emerging evidence that these fluctuations differ in clinical populations compared to healthy controls. However, the mechanisms that govern the dynamics of resting-state connectivity at different time scales are still poorly understood. Monitoring these dynamics in real-time enables assessment of data quality and sensitivity as intra-scan non-stationarity of connectivity can compromise the detection of RSNs in single subjects. Real-time monitoring of these dynamics is not only expected to improve consistency of data quality in clinical research studies, but will also contribute to our understanding of the neurophysiological mechanisms underlying the resting-state dynamics.

Seed-based correlation analysis (Van Dijk et al., 2010) and spatial independent component analysis (ICA) (Calhoun et al., 2001) are the principal tools to map functional connectivity, which have been shown to provide similar results (Van Dijk et al., 2010; Erhardt et al., 2011). Seed-based connectivity measures have been shown to be the sum of ICA-derived within- and between-network connectivities (Joel et al., 2011). ICA also performs spatial filtering, which enables segregation of spatially overlapping components. Seed-based techniques are sensitive to the choice of the seed regions (Cole et al., 2010a). On the other hand, source separation with ICA is sensitive to the selection of the model order, which is *a priori* unknown and necessitates dimensionality estimation approaches, such as the minimum description length (MDL), Bayesian information criterion (BIC), and Akaike’s information criterion (AIC) (Calhoun et al., 2001; Li et al., 2007). Furthermore, automated ordering of ICA components to enable consistent identification of RSNs is not yet feasible and source separation with ICA in individual subject data is limited by the contrast-to-noise ratio of the signal fluctuations and aliasing of cardiac- and respiration-related signal fluctuations. Seed-based correlation analysis surpasses ICA in detecting resting-state connectivity, but it requires regression of confounding signals, which typically include the six parameters of motion correction and their derivatives, and the average signal from up to three brain regions (whole brain over a fixed region in atlas space, ventricles, and white matter in the centrum semiovale). Regression of these signals is computationally intensive and may remove RSN signal changes that are temporally correlated with confounding signals.

The measurement of functional connectivity in the resting state has been limited, in part, by sensitivity and specificity constraints of current fMRI data acquisition methods. Echo-planar imaging (EPI) methods necessitate long scan times and detection of resting-state signal fluctuation suffers from temporally aliased physiological signal fluctuation, despite ongoing efforts to develop post-acquisition correction methods (Glover et al., 2000; Deckers et al., 2006; Beall and Lowe, 2007; Behzadi et al., 2007). Movement during the fMRI acquisition is a major confound for resting-state connectivity studies obscuring networks as well as creating false-positive connections (Satterthwaite et al., 2012; Van Dijk et al., 2012) despite state-of-the-art motion “correction” in post-processing. Distinction of BOLD contrast-based resting-state activity and of confounding physiological signal fluctuations has been shown to benefit from multi-echo acquisition. This approach not only increases BOLD sensitivity (Posse et al., 1999), but was also found to enable differentiation of BOLD contrast-based resting-state activity and of confounding physiological signal fluctuations (Kundu et al., 2012; Wu et al., 2012). However, multiple echo acquisition reduces temporal resolution and/or volume coverage, which have limited practical applications (Posse, 2012).

Recent advances in high-speed fMRI method development that enable un-aliased sampling of physiological signal fluctuation have considerably increased sensitivity for mapping task-based activation and functional connectivity, as well as for detecting dynamic changes in connectivity over time (Feinberg et al., 2010; Posse et al., 2012; Smith et al., 2012). High temporal resolution fMRI improves separation of RSNs using data driven analysis approaches (Smith et al., 2012) and may facilitate detecting the temporal dynamics of RSNs at much higher frequencies (up to 5 Hz) than detectable with traditional resting-state fMRI (Boubela et al., 2013; Boyacioglu et al., 2013; Chu et al., 2013; Lee et al., 2013). The development of ultra-high-speed fMRI methods with temporal resolution on the order of 100 ms or less has focused on echo-volumar imaging (EVI) (Rabrait et al., 2008; Witzel et al., 2008; van der Zwaag et al., 2009), inverse imaging (InI) (Lin et al., 2006, 2008, 2010), and MR encephalography (MREG) using highly undersampled projection imaging (Grotz et al., 2009), and fast volumetric imaging based on single-shot 3D rosette trajectories (Zahneisen et al., 2011). However, these single-shot methods are associated with degradation of spatial resolution and image uniformity. The recent development of simultaneous multi-slice (SMS) EPI using parallel imaging with blipped CAIPI acquisition increases temporal resolution without the *√ R* penalty incurred when using conventional parallel imaging methods, while maintaining acceptable image quality (Setsompop et al., 2012). Typical acceleration factors of eightfold are achievable using a 32 channel coil and faster acceleration has been shown in combination with in-plane parallel imaging (Moeller et al., 2010) and simultaneous echo refocusing (Feinberg et al., 2010; Chen et al., 2012). Recent advances in SMS-EPI enable up to 16-fold acceleration. Although acceleration is limited by RF power deposition (SAR), necessitating small flip angles, and image degradation at high acceleration factors due to increasing slice cross-talk and worsening g-factor (Moeller et al., 2010, 2012), SMS-EPI currently enables much higher spatial resolution compared to EVI. Furthermore, recent advances in RF pulse design, such as spatially periodic pulses, mitigate the RF power requirement for SMS EPI (Norris et al., 2011; Koopmans et al., 2013). We have recently introduced parallel imaging accelerated sequential multi-slab echo-volumar imaging (MEVI), which shortens the long EVI readout to achieve an image quality approaching that of EPI, and have demonstrated significant increases in BOLD sensitivity compared to EPI (Posse et al., 2012). This methodology enables ultra-high-speed real-time fMRI on conventional clinical 3 T scanners with 276 ms temporal resolution for whole brain acquisition and 136 ms temporal resolution for partial brain acquisition.

In the present study the *primary goals* were to characterize the sensitivity of MEVI for mapping major RSNs, comparing ICA and a novel real-time seed-based connectivity method that combines sliding-window correlation analysis with meta-statistics, and to map dynamic changes in resting-state connectivity at short time scales. The hypotheses for this novel seed-based connectivity approach are that: (a) resting-state connectivity can be measured at short time scales (seconds) and (b) averaging across short-term connectivity maps avoids the conventional artifact prone correlation across the entire scan. The *secondary goals* were to: (a) compare resting state and task-based fMRI in patients with neurological disorders for localizing sensorimotor and visual cortex in the vicinity of brain tumors and arterio-venous malformations, and to (b) to assess the feasibility of monitoring disease-related changes in functional connectivity in epilepsy. Localization of eloquent cortex adjacent to brain lesions is of critical value in presurgical planning and decision-making. Mapping of RSNs using fMRI has been suggested as an alternative to task-based fMRI, however, the utility for presurgical planning is still under investigation (Liu et al., 2009; Zhang et al., 2009; Mannfolk et al., 2011; Stufflebeam et al., 2011). The *tertiary goal* was to characterize regional differences in the cardiac-related cerebro-vascular pulsation in the healthy controls and in the patients with brain tumors, arterio-venous malformations, and epilepsy. Virtually all fMRI studies so far have sought to remove physiological signal fluctuations due to cardiac and respiration using model-based retrospective deconvolution methods (Glover et al., 2000). Ultra-high-speed fMRI enables direct observation of cardiac pulsation and its harmonics, which may carry important functional information that distinguishes healthy from diseased tissue vasculature.

## Materials and Methods

### Equipment

Data were collected on a clinical 3 T scanner, MAGNETOM Trio, A Tim System (Siemens Healthcare, Erlangen, Germany) equipped with MAGNETOM Avanto gradient system and 12-channel array receive-only head coil. A 32 channel coil became available during the last months of the study. Pulse and respiration waveforms were recorded with 1 kHz sampling rate using an MP150 data acquisition system and Acknowledge software 4.3 (Biopac Inc., Goleta, CA, USA). Reconstructed 2D images were exported from the scanner reconstruction computer via the scanner host computer to an external Intel Xeon E5530, six core, 2.4 GHz workstation for reconstruction of the third spatial dimension and real-time fMRI analysis, which were integrated into our custom TurboFIRE real-time fMRI software version V5.12.3.11.4.2 (Posse et al., 2001, 2012).

### Subjects

Nine healthy male and female subjects aged 21–50 years and eight patients with neurological disorders participated after giving institutionally reviewed informed consent.

#### Brain tumor

*Patient 1* was a 30-year old male with a low-grade right frontal lobe lesion associated with epilepsy and motor impairment, which was radiologically diagnosed as a low-grade glioma. The routine EEG demonstrated C4 (right central) epileptiform spikes. His seizures consist of an initial numbness and tingling sensation in the left arm and leg, followed by stiffening and jerking movements of the left side of the body. He failed treatment with oxcarbazepine, phenytoin, topiramate, and lorazepam. There was no obvious involvement of the primary motor cortex, based on the MEG motor and somatosensory responses, and the structural MRI. High-speed 3D short TE MR spectroscopic imaging (MRSI) using proton-echo-planar-spectroscopic-imaging (PEPSI) (Posse et al., 2007) showed increased Choline, reduced *N*-acetyl-aspartate (NAA), and strong lipid resonances, suggesting an oligodendroglioma (Posse et al., 2013). Intraoperative assessment confirmed a high lipid content. Postsurgical histology classified the tumor as an oligodendroglioma.

*Patient 2* was a 38-year old female with a 1.5-year history of headaches. The clinical MRI showed loss of gray-white matter differentiation with multiple areas of gyral expansion in the left superior frontal gyrus and in the left parietal lobe, which were suspected to be a primary glial tumor, such as multiple oligodendroglioma or multiple astrocytic tumors. High-speed 3D short TE MRSI using PEPSI (Posse et al., 2007) showed only a slight increase in Choline and slight reduction of *N*-acetyl-aspartate (NAA). The patient remained under observation. A biopsy performed a year later in the T_2_ hyperintense left parietal lesion revealed disease progression. The histological interpretation was infiltrating grade 2 astrocytoma.

#### Arterio-venous malformation

*Patient 3* was a 44-year old male with a two and a half year history of complex partial seizures and progressive right lower extremity weakness, who on imaging studies was found to have a Spetzler–Martin grade III arterio-venous malformation in the left fronto-parietal area. Cerebral angiography demonstrated a dense nidus with feeders from anterior, middle, and posterior cerebral arteries with early drainage into the superior sagittal sinus without significant deep drainage. Because of its location in the eloquent cortex, definitive treatment, either by surgery or endovascular means was not recommended. His seizures followed a Jacksonian-March pattern: starting from his right foot and marching up. The frequency of seizures at the time of testing was variable, ranging from daily to weekly, despite treatment with multiple anti-epileptic medications. The patient’s interictal EEG did not contain epileptiform abnormalities. He is on multiple anti-epileptic medications and his seizure control remains a challenge.

*Patient 4* was a 24-year old male with new onset of seizures with vivid visual aura who on workup was found to have a vascular lesion in the right occipital region. He described his aura as colors of rainbow that started in the center of the visual field and quickly shifted to the left hemifield followed by a generalized tonic-clonic seizure. Cerebral angiography demonstrated a clear hypervascular nidus without early venous drainage to qualify for an AVM. He underwent a surgical resection, which showed an arterial venous malformation with multiple thrombosed cortical veins. He is currently been weaned off his anti-epileptic medications and remains seizure free.

#### Temporal lobe epilepsy

*Patient 5* was a 53-year old male who had temporal lobe epilepsy with right mesial temporal lobe sclerosis and complex partial seizures preceded by deja vu, sometimes progressing to a secondarily generalized seizure. Epilepsy monitoring during withdrawal of anti-epileptic medication demonstrated seizures electrographically localized to the right anterior temporal area, and all interictal epileptiform activity similarly arising from the right anterior temporal area (F8 maximal). FDG-PET scanning demonstrated right mesial temporal hypometabolism and MEG interictal epileptiform activity localized to the left anterior temporal lobe in a distribution typical for mesial temporal epilepsy. He underwent temporal lobe resection and remains seizure free.

*Patient 6* was a 12-year old female who had had complex partial seizures with left temporal FDG-PET hypometabolism and seizures lateralized to the left hemisphere on non-invasive epilepsy monitoring. Invasive monitoring demonstrated seizure onset in the left temporal mesial area.

#### Cortical epilepsy

*Patient 7* was a 27-year old female with right posterior temporal lobe epilepsy. She suffered simple and complex partial seizures. FDG-PET demonstrated right posterior temporal hypometabolism and EEG and MEG localized interictal epileptiform spikes to the right occipital area. MRI demonstrated a right occipital area of cortical dysplasia, consistent with the patient’s left homonymous hemianopia.

*Patient 8* was a 50-year old male who had a left hemispheric localized cortical dysplasia associated with epilepsy and a prior history of stroke and transient ischemic attack. The MRI showed gyral expansion in the left frontal lobe with abnormal T_2_ signal extension through the cortical mantle to the ventricular margin. The morphology suggests focal transmantle cortical dysplasia with balloon cells. Single voxel MR spectroscopy and MRSI demonstrated elevated choline, consistent with focal cortical dysplasia. At the time of testing he had failed to gain complete seizure control despite trying multiple anti-epileptic medications.

### Data acquisition

Resting-state fMRI data were acquired using a MEVI pulse sequence with flyback along the *k_z_*-direction, which was described in Posse et al. (2012). Briefly, multiple adjacent slabs were excited sequentially in a single TR and encoded using repeated EPI modules with interleaved phase encoding gradients, fourfold acceleration using partial parallel imaging (GRAPPA), 6/8 partial Fourier encoding, and oversampling along the slab-direction. The reconstruction pipeline used distributed computing across the scanner using the ICE environment for in-plane (*k_x_*, *k_y_*) reconstruction and the external workstation using TurboFIRE (Posse et al., 2001) for reconstruction of the third dimension (*k_z_*) as described in Posse et al. (2012). The time delay from acquisition to display of reconstructed images was less than a TR. MEVI data were acquired using the following parameters:

- Four-slab EVI/MEVI4: TR: 276 ms, TE_eff_: 28 ms, α: 10°, four slabs in AC/PC orientation, interleaved acquisition order, slab thickness: 24 mm, inter-slab gap: 10%, matrix per slab: 64 × 64 × 8, Field of View (FOV) per slab: 256 × 256 × 32 mm^3^, reconstructed isotropic voxel dimensions: 4 mm, 27 slices, scan time: 5 min and 15 s using 1100 scan repetitions.
- Two-slab EVI/MEVI2: TR: 136 ms, TE_eff_: 28 ms, α: 10°, two slabs in AC/PC orientation, slab thickness: 42 mm, inter-slab gap: 10%, matrix per slab: 64 × 64 × 8, FOV per slab: 256 × 256 × 48 mm^3^, reconstructed voxel dimensions: 4 × 4 × 6 mm^3^, 13 slices, scan time: 5 min and 16 s using 2200 scan repetitions.

The 32 channel coil was used in one healthy control studied with MEVI2, in two of the five patients studied with MEVI2, and in two of the three patients studied with MEVI4, where one patient was scanned using both methods. *Patient 7* was studied using MEVI2 with the 12-channel coil and eight repetitions of 2.5 min scan time.

For comparison, resting-state scans in one healthy control was performed with multi-echo EPI using six TEs ranging from 5.8 to 49 ms, TR: 2 s, FOV 256 mm, spatial matrix, threefold GRAPPA acceleration, 6/8 partial Fourier encoding, 3.6 mm slice thickness, 10% slice gap, 168 scan repetitions, and 5 min 55 s scan time. Multi-echo data were combined using weighted echo averaging (Posse et al., 1999).

Task-based fMRI in patients was performed with multi-echo EPI using 10 TEs ranging from 5.8 to 82 ms, TR: 3 s, FOV 256 mm, spatial matrix, threefold GRAPPA acceleration, 6/8 partial Fourier encoding, 3.6 mm slice thickness, 10% slice gap, 56 scan repetitions, scan time: 3 min 12 s. Multi-echo data were combined using weighted echo averaging (Posse et al., 1999).

Structural imaging was performed using high-resolution Turbo-Spin-Echo and multi-echo MP-RAGE scans. Diffusion tensor MRI was performed using TR/TE: 9 s/84 ms, 35 gradient directions, *b*-values: 0 and 800 s/mm^2^, voxel size: 2 × 2 ×2 mm^3^, and scan time: 5 min 42 s.

### Resting state and activation tasks

Resting-state scans were performed during eyes open condition. Subjects were instructed to relax, clear their minds, and fixate on a crosshair presented on a computer screen.

The block-design auditory-gated visual-motor activation task consisted of eyes open in the lit scanner environment versus eyes closed, and simultaneous 2 Hz right hand index finger tapping versus rest. Subjects were asked to tap with maximum extension of the index finger. Covert word generation was performed in response to presentation of single letters. The task duration was 12 s and the interstimulus interval was 18 s. Five blocks of task activation were performed. Subjects were instructed to attend to each task with a constant effort across scans. Paradigm presentation was programed using ePrime software (Psychology Software Tools, Inc., Pittsburgh, PA, USA). Visual stimulation was provided using an in-house built MR compatible projection system. Auditory stimulation was delivered using an MR compatible headset (Avotek Inc., Stuart, FL, USA). An in-house developed button-response device (MIND Research Network, Albuquerque, NM, USA) was employed to monitor motor task execution.

### Data analysis

#### Retrospective ICA analysis

Spatial ICA was performed using the GIFT software package v1.3i^1^. Preprocessing using SPM8^2^ consisted of motion correction, coregistration with the EPI.mni template and spatial normalization to ensure consistent multi-session and/or multi-subject analysis. Spatial interpolation and Gaussian smoothing (6 × 6 × 6 mm^3^) was applied. The ICA algorithm used throughout was FastICA introduced by Hyvarinen and Oja (1997), since it had previously been shown to be more robust and computationally efficient compared with the competing alternative approaches for fMRI data analysis (Mutihac and Van Hulle, 2004). The settings used for all data sets were the following: epsilon: 10^−6^, maximum number of iterations: 1024, maximum number of fine-tuning sessions: 64, using tanh as the non-linear transfer function, sample size: 1, deflation mode, stabilization: on, and pow3 as “g” function. In order to estimate the data subspace (model selection), MDL was applied to the raw data. Alternatively, heuristically settings of 64 and 128 estimated number of independent latent sources, respectively, were investigated in view of detecting as many as possible default networks irrespective of any data model selection criteria. The validation of ICA decomposition was carried out by running ICASSO^3^ for each subject, so that the most stable directions were selected after statistical resampling (bootstrap) of the raw data. Principal component analysis (PCA) was used for prewhitening based on singular value decomposition. A *Z*-threshold of 1.2 was used to map independent components (ICs). The maximum *Z*-scores in each component was measured. ICs representing RSNs were identified by visual inspection in reference to the MNI brain atlas using spatial selection criteria described for 7 RSN categories and 28 components identified as RSNs in Allen et al. (2011). RSNs were further identified by slowly modulated signal time courses that were well above noise level. The power spectral density (PSD) estimate was computed by means of Welch’s overlapped segment averaging estimator implemented in MATLAB.

A time-frequency analysis of the time courses of RSN identified in two-slab EVI data was performed using the spectrogram function in MATLAB with a 28.6-s window for the FFT and 24.3 s overlap. The high-frequency limit of the RSN spectrum was measured using an amplitude threshold that was set at the level of the peaks of the high-frequency noise level outside of the cardiac and the respiratory bands.

#### Online seed-based sliding-window correlation analysis with meta-statistics

Real-time fMRI analysis was performed using TurboFIRE (Posse et al., 2001). Data preprocessing included motion correction, spatial normalization into MNI space using the SPM99 EPI template (Gao and Posse, 2003), segmentation of the MNI atlas space into 144 brain regions in reference to the Talairach Daemon Database that segregated left and right hemispheric regions (Zheng et al., 2013), and spatial smoothing using an 8 × 8 × 8 mm^3^ Gaussian filter. Signal fluctuation due to cardiac pulsation and respiration was suppressed using a 4-s time domain moving average filter (Lin et al., 2011). Detrending of confounding signal changes using weighted subtraction of multiple ROI time courses from white matter, CSF, and the entire brain was implemented as an option. Six single voxel seed locations were selected in reference to the MNI coordinates of the peak activations in six of the seven principal RSN categories reported in Allen et al. (2011):

- Auditory RSN (IC17): left superior temporal gyrus (BA22), coordinate: −51, −18, 7
- Sensorimotor RSN (IC7): left precentral gyrus (BA4), coordinate: −52, −9, 31
- Visual RSN (IC64): bilateral lingual gyrus (BA17, 18), coordinate: 1, −71, 13
- Default mode RSN (IC50): bilateral precuneus (BA7), coordinate: 1, −64, 43
- Attention RSN (IC34): left inferior parietal lobule (BA40), coordinate: −47, −57, 39
- Frontal RSN (IC42): right inferior frontal gyrus (BA45), coordinate: 50, 23, 2

The signal time course within each seed region was used as input to dynamic reference vector modeling (Gao and Posse, 2004), which was adapted to bypass convolution with the hemodynamic response function. Seed-based sliding-window correlation analysis was combined with a meta-statistics approach that employs an efficient running variance algorithm (Welford, 1962) across dynamically updated correlation maps to generate cumulative meta-statistics maps of the mean and the standard deviation. The sliding-window width (*N*_w_) was 4, 8, 28, 52, 105, 210, or 420 scans, i.e., 1, 2, 4, 8, 15, 30, or 60 s, respectively. The initial 50 scans were discarded (*N*_d_). Correlation values were threshold with correction for degrees of freedom as described in eq. 13 in Bandettini et al. (1993) using a cross-correlation threshold of 0.52. Meta-statistics were computed at each TR starting at (*N*_d_ + *N*_w_) and the final meta-statistics maps were used for individual and group analysis. The final meta-statistics maps were segmented into 144 brain regions based on the modified Talairach Daemon database. Cross-correlation coefficients between the six seed ROI time courses were computed at each TR.

#### Offline processing of seed-based connectivity results

A metric of functional network connectivity (FNC) was created by spatially averaging the meta-statistics maps within each brain region. The group average across nine subjects of the *intra-network* FNC within each of six major seeded RSNs was computed using the following subset of brain regions (Allen et al., 2011):

- Auditory RSN: left and right BA22, BA24
- Sensorimotor RSN: left and right BA2, BA4, BA6
- Visual RSN: left and right BA17, BA18
- Default mode RSN: left and right BA7, BA10, BA23, BA31, BA32, BA39
- Attention RSN: left and right BA8, BA40
- Frontal RSN: left and right BA22, BA44, BA45

Signal time courses from the six seed regions were extracted at each TR to represent RSN time courses. A matrix of cross-correlation coefficients between the different RSN time courses was computed as a metric of *inter-network* FNC at 4 s intervals. Time averaged matrices were computed across entire scans. The rows of the inter-network FNC matrix were averaged to obtain a metric of *global* FNC for each seed region. Group averages of the inter-network and global FNC across nine subjects were computed.

#### Cardiac pulsatility

The time course of ICs with cardiac-related signal pulsation, measured in nine healthy controls and in seven patients who underwent resting-state fMRI using MEVI2, was analyzed in a beat-by-beat manner using an automatic delineator method that identifies fiducial points of the pulsation waveform (Li et al., 2010) to enable coherent time averaging of the pulsation waveforms in the presence of heart rate variability. The averaged waveforms were replicated 128 times and Fourier transformed to create power spectra of cardiac-related pulsation. The peak amplitudes at the cardiac frequency, the first harmonic, and the second harmonic were measured. The ratio *R* of the amplitude of the peak at the cardiac frequency with respect to the amplitude of the first harmonic was computed.

#### Diffusion tensor imaging

DICOM images were converted to NIFTI format using the MATLAB toolbox MRIconvert. Eddy current correction was performed in FSL using the FDT Diffusion toolbox. Brain masking was applied to exclude artifacts outside the brain. DTI analysis with tractography was performed using MedINRIA software^4^. Manually defined seed ROIs in the motor pathways were used for fiber tracking.

#### Statistical analysis

The TurboFIRE data output was post-processed using custom PERL scripts and spreadsheets, and standard MATLAB toolboxes. Statistical analysis was performed using a two-tailed heteroscedastic Student’s *t*-test.

## Results

### Resting-state fMRI in healthy controls using ICA

**Figure 1:**
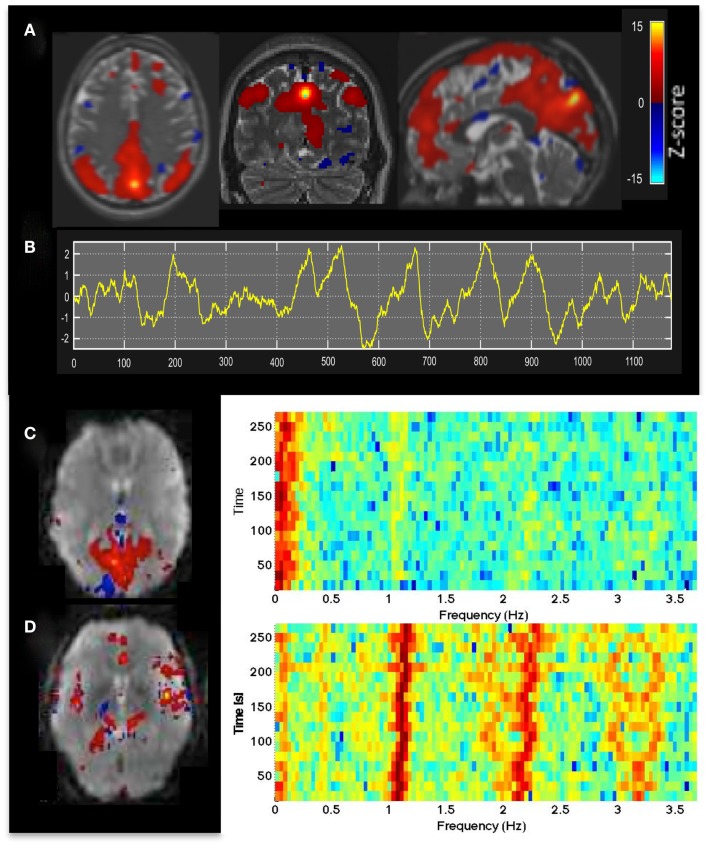
**(A)** Resting-state fMRI in a healthy control using whole brain MEVI4 with TR: 276 ms. The spatial ICA map with *Z*-scores up to 15 shows a clearly delineated default mode RSN. **(B–D)** ICA-based mapping of RSNs and cardiac pulsatility using MEVI2 with TR: 136 ms. **(B)** Slowly varying signal changes well above noise level (*z*_max_ > 10) distinguish **(C)** RSNs from **(D)** cardiac-related signal pulsation. The corresponding spectrograms display **(C)** the dynamically fluctuating low-frequency power spectrum of the RSN and **(D)** the first and second harmonics of the cardiac pulsation.

Independent component analysis analysis of MEVI2 and MEVI4 data showed clear delineation of major RSNs (Figure 1A) described in Allen et al. (2011), and separation of multiple ICs showing cardiac- and respiration-related signal pulsation. ICs with RSNs were characterized by slowly varying signal time courses with high contrast-to-noise-ratio well above noise level (Figure 1B) and small contamination from cardiac- and respiration-related signal pulsation (Figure 1C). Cardiac-related signal pulsation was resolved on a beat-by-beat basis in synchrony with peripheral pulse recording (Figure 1D). The corresponding ICs mapped cardiac signal pulsation in insular cortex, cortical gray matter, brain stem, sagittal sinus, and ventricles. Respiration-related signal changes were detected at the edges of the imaging slabs. Brief head movements were clearly detected as separate ICs with spatial components located in orbital frontal cortex and at the edges of the brain.

Independent component analysis of 5 min 25 s scans collected in eight subjects using MEVI2 and the 12-channel coil separated on average 28.4 ± 7.2 ICs, which consisted on average of 11.5 ± 5.7 ICs corresponding to the major RSNs described in Allen et al. (2011). In some subjects multiple RSNs belonging to a particular category (e.g., some of the six sensorimotor RSNs described in Allen et al., 2011) were mapped into different ICs, but co-localized with RSNs belonging to other categories (e.g., the auditory RSN) within single ICs. As a consequence, the sum of RSN, cardiac, respiratory, and artifact ICs exceeded the number of total ICs. On average, 12.8 ± 4.9 RSNs were identified in these ICs with some of the ICs containing up to three different RSNs. In addition, 6.6 ± 3.3 ICs corresponded to cardiac pulsation, 4.6 ± 2.9 ICs corresponded to respiration-related signal changes, and 7.8 ± 4.9 ICs corresponded to artifacts related to head movement and to 1 Hz signal oscillations, predominantly at the edges of the slabs (Table 1). Maximum *Z*-scores ranged from 5.2 to 20.2 for attentional RSNs with other RSNs having maximum *Z*-score within this range. The average *Z*-score across all RSNs was 11.8 ± 0.7. ICA analysis of data collected in one subject using the 32 channel coil separated 42 ICs, of which 20 were related to RSNs, 10 were related to cardiac pulsation, 5 were related to respiration related signal changes, and 13 were related to head movement and artifacts, including coherent constant amplitude 1 Hz signal oscillation at the edges of the brain and in parietal cortex. *Z*-scores reached up to 32.2 for sensorimotor and attentional RSNs, and the average *Z*-score across all RSNs was 18.4. These results are consistent with the data collected in the patients (see below). Table 1 shows the results averaged across all nine subjects.

**Table 1:** **Source separation using ICA in healthy controls and patients**.

# Components		Total	RSN	Basal ganglia	Auditory	Sensori-motor	Visual	Default mode	Attentional	Frontal	Lesion	Sum/mean	Cardiac	Respiratory	Artifact
		IC	IC	RSN	RSN	RSN	RSN	RSN	RSN	RSN	RSN	RSN	IC	IC	IC
**CONTROLS**															
MEVI2	Mean	29.9	11.8	0.0	0.8	2.4	2.3	2.0	4.6	1.4		13.6	7.0	4.7	8.3
(*n* = 9)	SD	8.1	5.4	0.0	0.4	2.6	1.3	0.9	2.4	0.9		5.2		2.7	4.9
MEVI2*	*n* = 1	34.0	19.0	0.0	0.0	9.0	1.0	4.0	6.0	1.0		21.0	3.0	6.0	6.0
MEVI4*	*n* = 1	21.0	8.0	1.0	1.0	1.0	4.0	1.0	5.0	1.0		14.0	2.0	1.0	8.0
MEPI*	*n* = 1	33.0	14.0	0.0	1.0	1.0	4.0	2.0	5.0	3.0		16.0	2.0	3.0	15.0
**PATIENTS**															
MEVI2	Mean	38.6	16.6	0.0	1.4	2.4	3.4	3.2	5.8	1.6	1.8	19.6	7.6	5.2	11.4
(*n* = 5)	SD	13.4	7.8	0.0	0.9	1.7	2.1	2.2	2.5	1.3	2.5	9.1	4.4	7.8	3.4
MEVI4	Mean	26.0	7.0	0.0	1.3	1.0	2.7	2.0	4.3	1.7	0.0	13.0	4.0	9.0	6.0
(*n* = 3)	SD	12.1	5.3	0.0	0.6	1.0	2.1	1.0	2.5	2.1	0.0	8.9	1.7	6.2	7.8
MEVI2**	Mean	18.3	6.9	0.1	1.1	1.1	2.4	1.4	2.6	1.0	1.1	10.9	3.3	1.0	7.9
	SD	1.0	1.8		0.4	0.4	0.9	0.5	0.7	0.0	0.8	2.0	0.5	0.9	1.4
**MAXIMUM *Z*-SCORES: CONTROLS**															
MEVI2	Mean				13.0	16.0	13.0	12.7	12.1	11.6		13.1	17.3	13.6	15.3
(*n* = 5)	SD				2.2	8.4	4.0	3.4	2.8	1.5		3.0	2.0	3.8	6.0

Top: total number of ICs based on the MDL criterion, and number of detected ICs that correspond to RSNs, cardiac-related signal pulsation, respiration related signal changes, and artifacts (*same subject, **single subject, eight scan repetitions, 2.5 min per scan). Bottom: maximum Z-scores averaged across all ICs in a given category and across healthy controls. MEVI2 = partial brain two-slab EVI (TR: 136 ms), MEVI4 = whole brain four-slab EVI (TR: 276 ms), MEPI = multi-echo EPI (TR: 2 s).

The time-frequency analysis of signal fluctuations in RSNs measured with MEVI2 was performed in five subjects. The spectrograms (Figures 1C,D) displayed low-frequency components that had maximum power around 0.1 Hz and extended on average to a maximum frequency of 0.27 Hz (Table 2). Short-term fluctuations of this frequency range at short times scales (i.e., individual 24.3 s segments) were up to ±0.1 Hz. The range of measurable RSN frequencies was also limited by residual signal fluctuation due to respiration, which in some cases overlapped with RSN frequency components.

**Table 2:** **High-frequency cutoff of low-frequency resting-state signal fluctuations in healthy controls**.

Subject	Mean (Hz)	SD (Hz)
1	0.29	0.02
2	0.25	0.02
3	0.32	0.09
4	0.26	0.02
5	0.22	0.02
Mean	0.27	0.03
SD	0.04	0.03

### Sensitivity comparison MEVI2, MEVI4, and MEPI

In one healthy subject a sensitivity comparison was performed between MEVI2, MEVI4, and weighted averaged multi-echo EPI using the 12-channel coil and identical isotropic resolution (4 × 4 × 4 mm^3^). ICA analysis of the multi-echo EPI data separated 33 ICs, which were related to 16 RSNs (Table 1). RSNs measured with multi-echo EPI were mixed with aliased cardiac- and respiration-related signal pulsation and displayed spurious connectivity in white matter. ICs with predominantly cardiac (2) and respiratory (3) signal changes in these data were only identifiable based on their spatial localization in reference to the EVI results. The MEVI4 data in this subject displayed improved separation of cardiac- and respiration-related signal contamination, but smaller number of separated ICs (21), with 14 identifiable RSNs. The corresponding MEVI2 data showed further reduction of spurious connectivity in white matter, larger number of ICs (34) comparable to multi-echo EPI and larger number of identified RSNs (21).

In patients a corresponding trend was found: MEVI2 separated more ICs on average than MEVI4 (38.6 ± 13.4 versus 26.0 ± 12.1) with more ICs corresponding to RSNs (16.6 ± 7.8 versus 7.0 ± 5.3, *p* = 0.09). MEVI2 enabled identification of a larger number of RSNs (19.6 ± 9.1 versus 13.0 ± 8.9) and cardiac components (7.6 ± 4.4 versus 4.0 ± 1.7, *p* = 0.16) compared to MEVI4 (Table 1).

**Figure 2:**
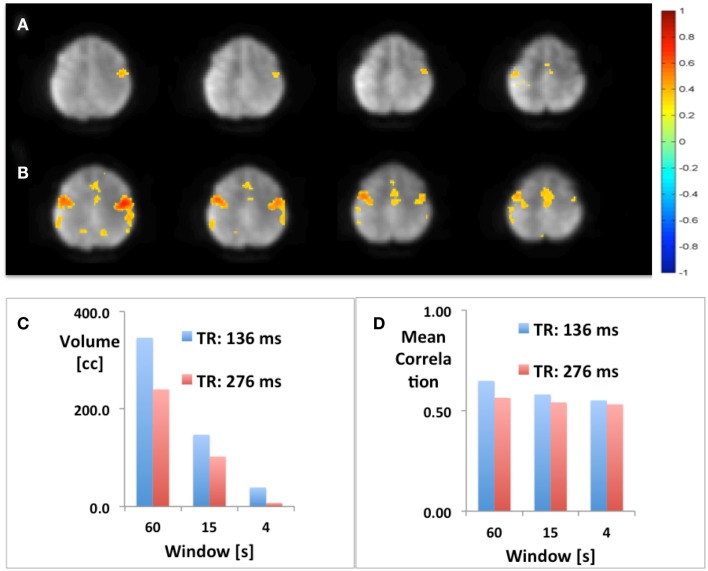
**Seed-based mapping with 4 s sliding window of the sensorimotor RSN comparing (A) MEVI4 (TR: 286 ms) and (B) MEVI2 (TR: 136 ms), which shows higher peak correlation and larger spatial extent of connectivity across the two-slab volume compared with MEVI4**. **(C)** The spatial extent of connectivity decreases strongly with sliding-window width, but it is larger with MEVI2 compared to MEVI4 at all three time scales. **(D)** The mean correlation is comparable across the range of time scales.

Using seed-based connectivity with meta-statistics (see below) MEVI2 yielded larger peak correlation coefficients and larger extent of connectivity across the two-slab volume compared with MEVI4 across a wide range of time scales from 4 to 60 s (Figure 2).

### Resting-state dynamics using seed-based connectivity with meta-statistics

**Figure 3:**
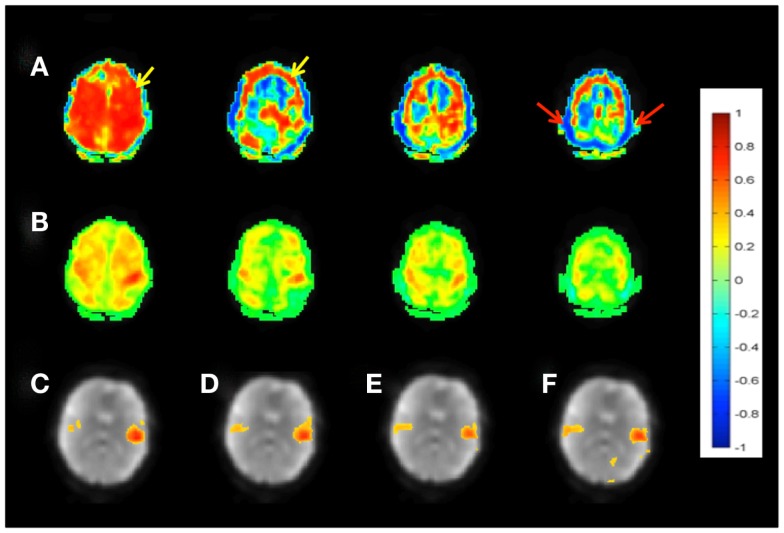
**Seed-based connectivity of the sensorimotor RSN measured with MEVI2 (TR: 136 ms)**. **(A)** Correlation across the entire 5 min scan without regression of confounding signal changes displays widespread artifacts (yellow arrow) and edge artifacts due to head movement (red arrows). **(B)** Sliding-window correlation analysis with meta-statistics using a 4-s sliding-window removes the artifacts and reveals the expected localization of the sensorimotor network in the mean meta-statistics map. **(C–F)** Seed-based connectivity of the auditory RSN shown as mean meta-statistics across the 5 min scan using sliding-window widths of **(C)** 15 s, **(D)** 4 s, **(E)** 2 s, and **(F)** 1 s.

The meta-statistics approach provided strong rejection of confounding signals from head movement, respiration, cardiac pulsation, and signal drifts (Figures 3A,B), without using regression of movement parameters and signals from white matter and CSF. The degree of rejection of confounding signals increased with decreasing sliding-window width, while mean correlation coefficients decreased only slightly. A window width of 60 s often provided considerable artifact suppression, but a 15-s window was preferred due to even more robust artifact suppression. The correlation coefficients in white matter and CSF using this approach were small, typically<0.2 (Figure 3B). Weighted subtraction of signals from white matter, CSF, and the entire brain did not result in consistent improvement of mapping the major RSNs.

**Figure 4:**
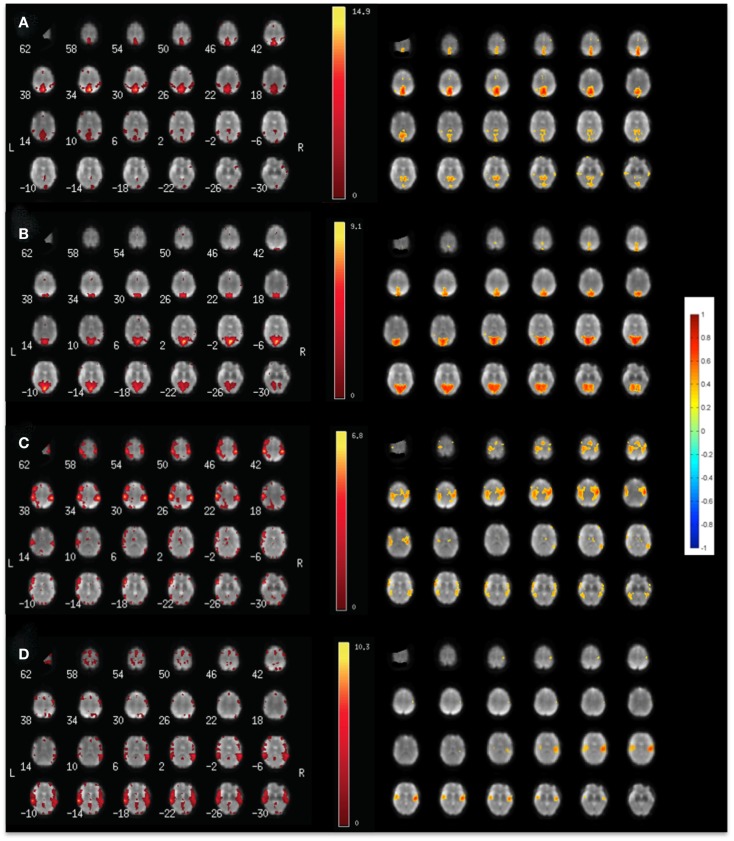
**Comparison of (left) ICA and (right) seed-based connectivity in a single subject for mapping (A) the default mode RSN, (B) the visual RSN, (C) the sensorimotor RSN, and (D) the auditory using MEVI2 at TR: 136 ms**.

Our data show high sensitivity for mapping intra- and inter-network connectivity at time scales as short as 4 s, which is consistent with the upper frequency range of signal fluctuation in major RSNs shown in Table 2. Interestingly, the auditory network displayed connectivity at time scales as short as 1 s with little decrease in mean correlation coefficient (Figures 3C–F). Using this meta-statistics approach RSNs were detected in tens of seconds. Some of the major RSNs, such as the DMN, the auditory network, and the visual network were often detectable in as little as 10–20 s. The localization and spatial extent of principal nodes of major RSNs using the seed-based analysis approach were comparable to the ICA results (Figure 4).

**Figure 5:**
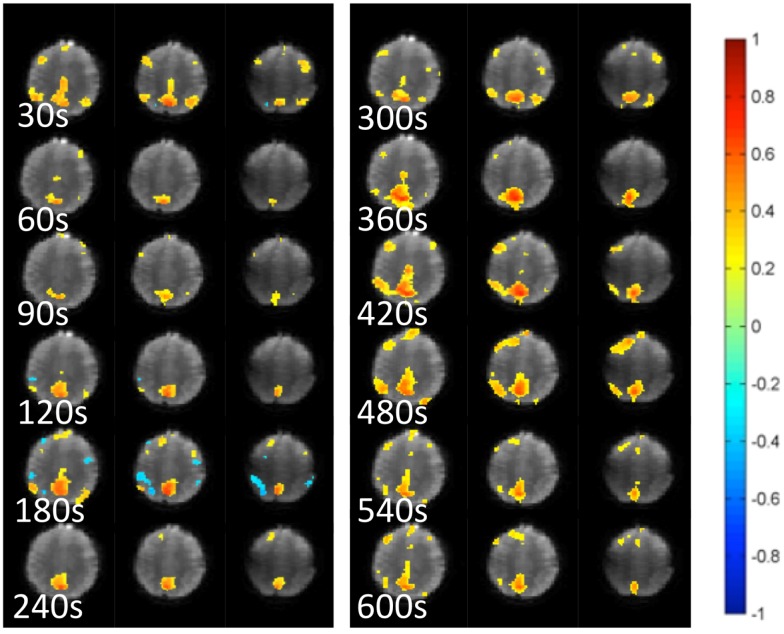
**Dynamic changes in temporal correlation within the default mode RSN measured at 30 s intervals using MEVI2 (TR: 136 ms) and sliding-window seed-based correlation analysis with 30 s window in a healthy control**.

**Figure 6:**
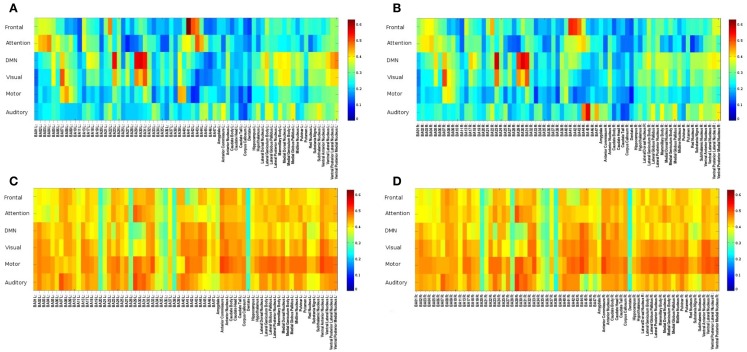
**Seed-based FNC between 6 seed regions and 144 brain regions using MEVI2 (TR: 136 ms) and meta-statistics averaged across nine healthy subjects at the end of the scan**. Spatial means of **(A)** left hemisphere meta-statistics means, **(B)** right hemisphere meta-statistics means, **(C)** left hemisphere meta-statistics standard deviations, and **(D)** right hemisphere meta-statistics standard deviations.

Mapping of dynamic changes in *intra-network* FNC revealed considerable differences in short-term fluctuations in different nodes of major RSNs. For example, the IPL region (BA39 + BA40) showed some of the strongest fluctuation within the DMN (Figure 5), consistent with a recent group ICA study (Allen et al., 2012). FNC between the 6 seeds and 144 brain regions averaged across an entire scan was predominantly positive and showed extensive connectivity across many brain areas. In general, major nodes of connectivity with higher short-term correlation were predominantly associated with lower standard deviation of short-term correlation as shown in Figure 6, which is an example of seed-based connectivity across 144 brain regions averaged across nine subjects using a 15-s sliding window. On the other hand, higher standard deviation was frequently measured in regions with lower short-term correlation. The default mode and the visual networks share a similar pattern of FNCs. There were notable right-left asymmetries in the meta-means maps: for example, FNC in the frontal network with BA45 showed the largest right side dominance (difference = 0.2), along with BA25, BA44, BA46, and BA47. The FNC in the attention network showed the largest asymmetry for BA39. The DMN showed the largest asymmetry in Medial Geniculum Body.

**Figure 7:**
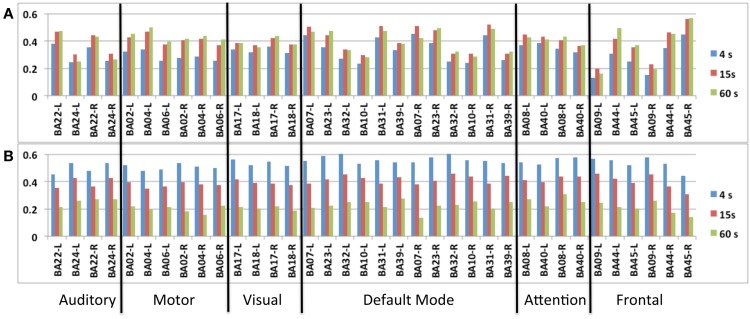
**Seed-based intra-network FNC at the end of the scan averaged across nine healthy subjects using MEVI2 (TR: 136 ms)**. Spatial means of the meta-statistics **(A)** means and **(B)** standard deviation in a subset of 18 selected Brodmann areas as a function of sliding-window width (4, 15, and 60 s).

A group analysis in nine subjects demonstrated that *intra-network* FNC measured using this sliding-window based meta-statistics approach yielded intra-network correlation values that were comparable in amplitude with previous studies using ICA (Figure 7) (Allen et al., 2012). Intra-network FNC in major nodes of six principal RSNs decreases moderately at 4 s sliding-window width compared to 15 and 60 s sliding-window widths (Figure 7A). Some of the strongest intra-network FNC was measured within the DMN. Consistent with previous studies, temporal fluctuations in intra-network FNC increased with decreasing sliding-window width (Figure 7B).

**Figure 8:**
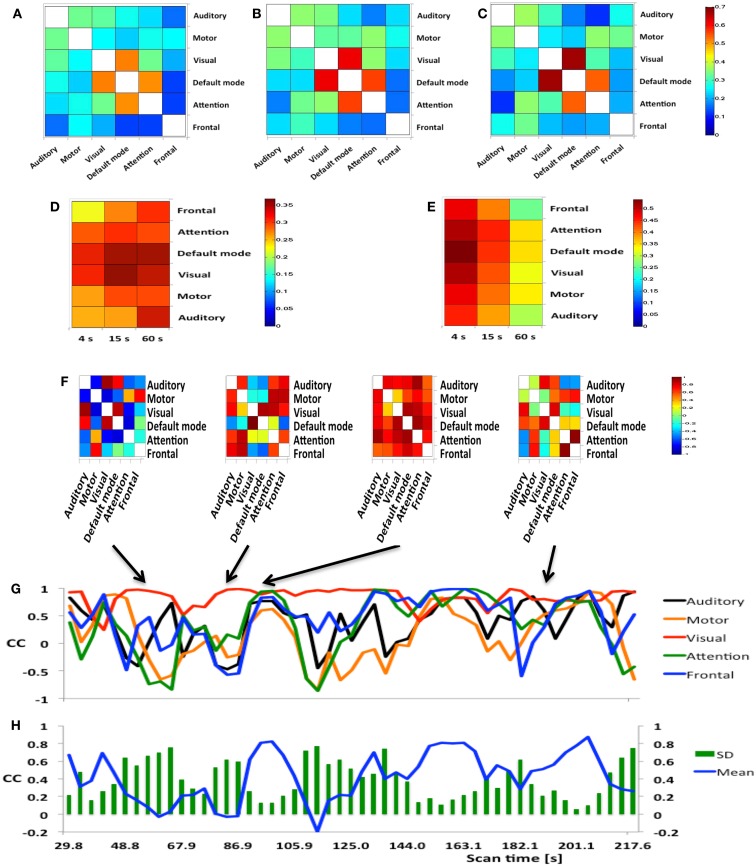
**(A–E)** Seed-based inter-network FNC averaged across nine healthy subjects using MEVI2 (TR: 136 ms) and **(F–H)**. Simulated real-time monitoring of inter-network FNC in a single subject using MEVI2 (TR: 136 ms) with the 12-channel coil and a 15-s sliding window. Subject average of the meta-statistics correlation coefficient matrix for six seeds at a time scale of **(A)** 4 s **(B)** 15 s, and **(C)** 60 s at the end of the scan. Group-averaged **(D)** mean and **(E)** standard deviation of global FNC for six seeds at time scales of 4, 15, and 60 s at the end of the scan. **(F)** Selected connectivity matrices for 15 s sliding windows at time points of low (64, 83 s), high (95 s), and intermediate (190 s) synchronization in a single subject. **(G)** Corresponding time courses of the correlations between the cuneus seed time course of the DMN and the seed time courses of five major task-positive RSNs within the sliding window. **(H)** Corresponding time courses of the mean and standard deviation of the correlation time courses in **(G)** as a metric of inter-network FNC.

A group analysis of *inter-network* FNC in nine subjects demonstrated mean correlation values comparable to previous studies using ICA (Figures 8A–C) (Allen et al., 2012). Some of the strongest inter-network FNC was measured between the DMN and the visual network, and between the DMN and the attention network. The inter-network connectivity increased moderately with increasing sliding-window width between 4 and 60 s. The *global* FNC averaged across nine subjects decreased moderately at 4 s sliding-window width compared to 15 and 60 s sliding-window widths (Figure 8D). Inline with the intra-network connectivity, temporal fluctuations in global FNC increased with decreasing sliding-window width (Figure 8E). The DMN displayed the strongest temporal fluctuation of global FNC at a time scale of 4 s.

Figure 8F shows a typical series of dynamic inter-network FNC matrices in a single subject for a seed in the DMN, which show both positive and negative FNC at short time scales (sliding-window width: 15 s) between the seed in the DMN and five seeds in task-positive RSNs. The corresponding five time courses of the short-term FNC within the sliding-window demonstrate rapidly changing correlations between positive and negative values (Figure 8G). The mean and the standard deviation across these correlation time courses show considerable fluctuation of inter-network coherence (Figure 8H). Similar short-term temporal dynamics of positive and negative FNC between the seed in the DMN and the five seeds in task-positive RSNs were observed in all subjects.

### Resting-state fMRI in patients with neurological disorders

Patients exhibited a greater number of RSNs on average compared to healthy controls (Table 1) due in part to the transition to the 32 channel coil. Spatial displacement of major RSNs and reduced connectivity within RSNs was mapped in the vicinity of brain tumors and vascular malformations. Unanticipated connectivity was also found in some of the patients. The following cases demonstrated noteworthy changes in functional organization.

**Figure 9:**
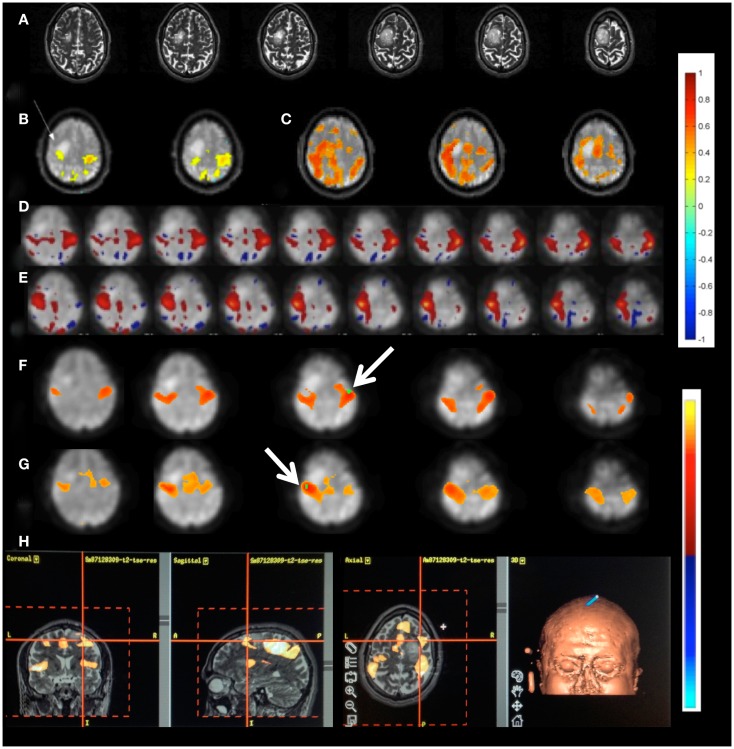
**Presurgical mapping in *patient 1 with right prefrontal low-grade oligodendroglioma***. **(A)** T2-weighted MRI. Task-based fMRI using MEPI (TR: 2 s). **(B)** Right hand finger tapping shows sharp delineation of eloquent cortex. **(C)** Left hand finger tapping shows diffuse activation in the vicinity of the tumor. Resting-state fMRI using MEVI2 (TR: 136 ms) and ICA shows **(D)** left sensorimotor cortex localization ICA (*z*_max_ = 7.9) consistent with task-activation in **(B,E)** right sensorimotor RSN mapping with showing more focal localization (*z*_max_ = 12). Seed-based analysis shows focal localization of the sensorimotor RSN consistent with ICA: **(F)** left motor seed and **(G)** right motor seed (arrows). **(H)** Sum of all seed-based resting-state networks in the vicinity of the tumor integrated into presurgical planning. Color scales for task-based correlation analysis and seed-based connectivity (top), and ICA (bottom) are shown on the right.

*Patient 1* with a frontal lobe brain tumor showed much stronger activation of motor cortex and extensive activation of non-motor areas adjacent to the tumor during left hand index finger tapping compared to right hand index finger tapping (Figure 9). This may reflect the increased effort of left hand task execution, which the patient reported, and dysregulation of cerebro-vascular coupling within the edema around the tumor. By contrast, the sensorimotor RSNs measured in this patient showed comparable focal connectivity within both motor cortices. Interestingly, the sensorimotor RSN was separated into two lateralized subnets, which suggests reduced functional connectivity within the sensorimotor RSN due to the tumor. In this patient we also illustrate the integration of RSN maps into the StealthStation neuronavigation system (Medtronics, MN, USA) for presurgical planning using the sum of all RSNs in the vicinity of the tumor (Figure 9H).

Functional connectivity mapping in *patient 2* with a posterior temporal lobe tumor showed decreased connectivity in and adjacent to the lesion in DTI-based fiber tracking and in the default mode RSN. Interestingly, the sensorimotor RSN was not detected with ICA although the other major RSN were present and task-based fMRI clearly localized sensorimotor cortex. Seed-based connectivity using seed locations based on motor activation that was detected in task-based fMRI mapped the sensorimotor RSN.

**Figure 10:**
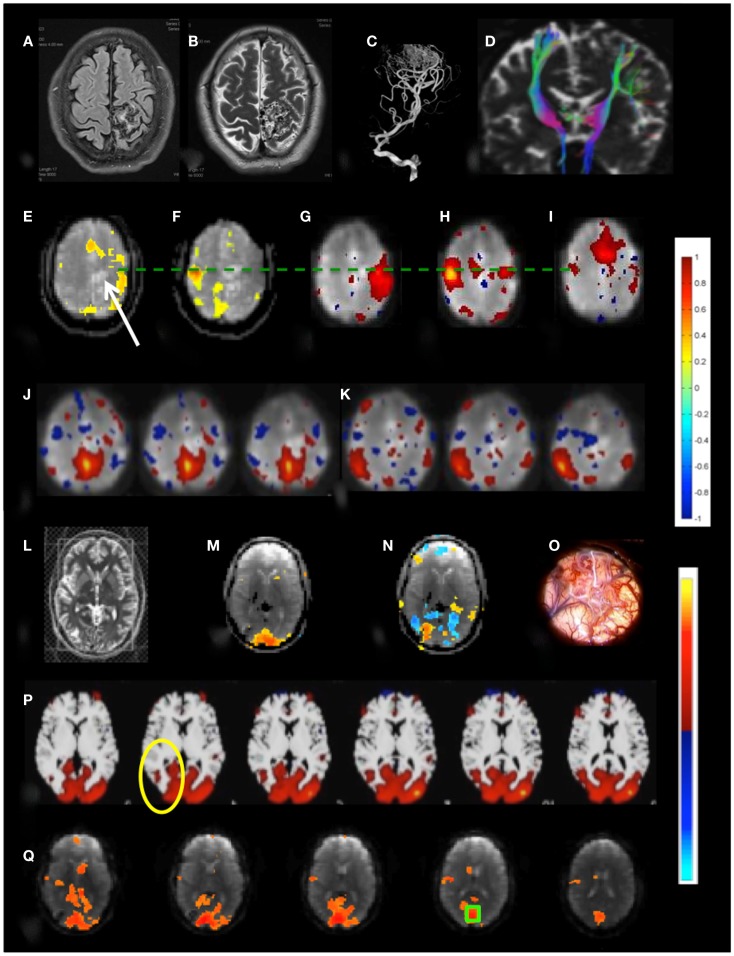
**Presurgical mapping in two patients with arterio-venous malformations (AVM)**. *Patient 3 with left parietal AVM*: **(A)** T1-weighted MRI, **(B)** T2-weighted MRI, **(C)** MR-angiogram, **(D)** DTI-based fiber tracking show distortion of motor pathways. Task-based fMRI using MEPI (TR: 2 s): **(E)** right hand finger tapping shows distributed activation around the lesion (indicated by arrow) beyond the left motor cortex and in the supplementary motor area and **(F)** left hand finger tapping shows focal localization of right motor cortex. Resting-state fMRI using MEVI2 (TR: 136 ms) and ICA segregates the sensorimotor RSN into three subnetworks with focal localization of motor areas: **(G)** a right sensorimotor RSN (*z*_max_ = 7.9), **(H)** a left sensorimotor RSN (*z*_max_ = 8.9), and **(I)** a supplementary motor area RSN (*z*_max_ = 9.4). ICA also segregates the default mode RSN into two subnetworks **(J,K)** that do not extend into the left parietal cortex (*z*_max_ = 6.2 and 8.5, respectively). *Patient 4 with right occipital AVM*: **(L)** T2-weighted MRI. Task-based fMRI using MEPI (TR: 2 s). **(M)** Visual stimulation does not activate the lesion and **(N)** imagination of the experience of the “aura” associated with epilepsy activates and deactivates areas at the rim of the lesion. **(O)** Intraoperative image. **(P)** Resting-state fMRI using MEVI2 (TR: 136 ms) and ICA shows a visual RSN that does not extend into the AVM consistent with visual stimulation (*z*_max_ = 12.6). **(Q)** Seed-based functional connectivity of the visual RSN with a seed in BA 17 (green box) does not show visual eloquence within the AVM. Color scales for task-based correlation analysis and seed-based connectivity (top), and ICA (bottom) are shown on the right.

*Patient 3* with a temporal lobe AVM exhibited extensive recruitment of brain regions in the vicinity of the AVM during right finger tapping, which may reflect the considerably increased effort of task execution compared to left hand finger tapping and dysregulation of cerebro-vascular coupling in the vicinity of the AVM (Figures 10A–K). The resting-state sensorimotor network showed a complete disconnection on the side of the AVM, resulting in the detection of with three separate RSNs in the left and the right sensorimotor cortex and the supplementary motor area.

*Patient 4* with an occipital lobe AVM displayed asymmetrical activation in visual cortex during visual stimulation that excluded the rims of the AVM (Figures 10L–Q). Interestingly, a visual imagery task that involved imagining the “aura” resulted in a complex activation pattern along the rims of the AVM, suggesting that these regions may be involved in the visual aura associated with the seizure. The major visual RSN, which was detected both with ICA and seed-based correlation analysis, excluded the rims of the AVM. However, a seed placed in the AVM revealed extensive connectivity with secondary visual cortex.

**Figure 11:**
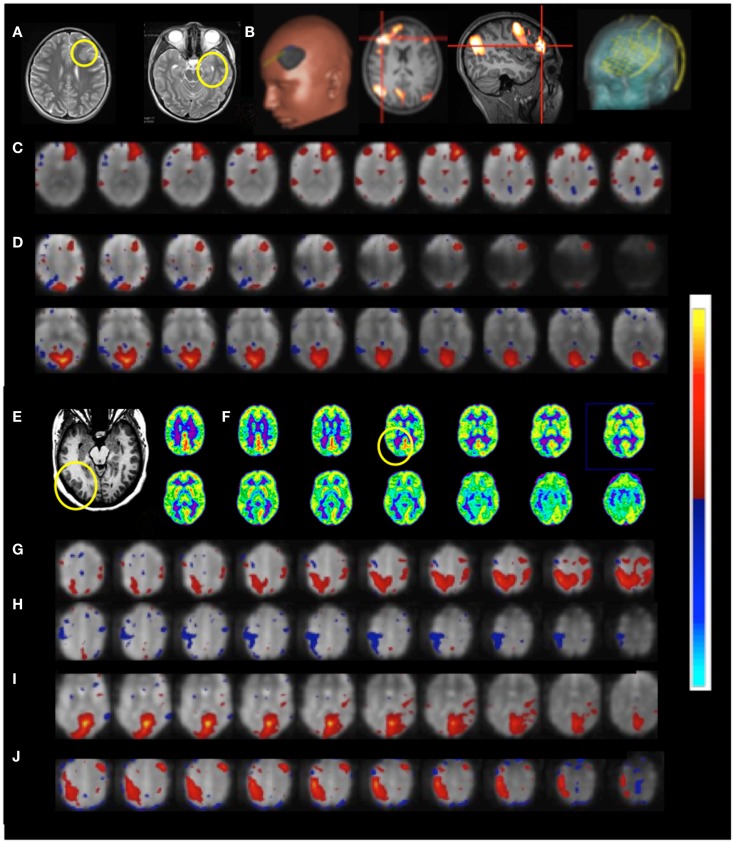
**Presurgical mapping in two patients with epilepsy Resting-state fMRI using MEVI2 (TR: 136 ms) and ICA**. *Patient 6 with temporal lobe epilepsy*. **(A)** The T_2_-weighted MRI shows hyperplasia in anterior left frontal cortex and left mesial temporal lobe sclerosis. **(B)** Presurgical planning using resting-state networks encompassing language areas and the area of dysplasia. ICA shows **(C)** a resting-state network encompassing the area of dysplasia (*z*_max_ = 15.6) and **(D)** abnormal connectivity between the area of dysplasia and the visual RSN (*z*_max_ = 9.0). *Patient 7 with cortical epilepsy*. **(E)** The T_1_-weighted MRI shows cortical thickening in the right posterior temporal lobe (yellow circle) **(F)** FDG-PET shows hypometabolism in this region (yellow circle). ICA shows dynamic RSN changes in consecutive scans. **(G)** An unanticipated RSN emerges in right posterior parietal and temporal cortex during scan 4 (*z*_max_ = 15.0). **(H,I)** In scan 6 the visual RSN displays spatial asymmetry that excludes the right posterior temporal lobe and exhibits negative correlation with the right motor and posterior parietal cortex (*z*_max_ = 17.3). **(J)** The unanticipated RSN in right posterior parietal and temporal cortex extends into inferior regions during scan 8 (*z*_max_ = 16.3). The color scale for ICA is shown on the right.

*Patient 6* with temporal lobe epilepsy exhibited hyperplasia in anterior left frontal cortex (Figures 11A–D), which in DTI-based fiber tracking shows reduced connectivity and, uncharacteristically for epilepsy, hypermetabolism in this region in the FDG-PET scan. Cortical recordings using an implanted electrode grid (Figure 11B) showed that this region was not the source of epileptic activity. The default mode RSN showed asymmetric connectivity in the frontal cortex. The attention RSN displayed spatial asymmetry as well, whereas the visual RSN displayed connectivity with the hyperplasia lesion. The lesion itself was also connected to other cortical regions, which was mapped in a separate IC. In this patient we also illustrate the integration of RSN maps into the StealthStation neuronavigation system (Medtronics, MN, USA) for presurgical planning.

Functional connectivity mapping in *patient 7* with cortical epilepsy revealed progressive changes in functional connectivity during eight consecutive resting-state scans (Figures 11E–J). In the third scan the visual RSN became spatially asymmetric. In the fourth scan a new RSN was detected that encompassed right posterior parietal and temporal cortex, a region that showed interictal spike activity in EEG and MEG. The visual RSN was spatially asymmetric and the sensorimotor RSN was not detected. In the fifth scan a spatially asymmetric visual RSN was detected again. In scan 6 the spatial asymmetry of the visual RSN increased, excluding the right posterior temporal lobe, and negative correlation with the right motor and posterior parietal cortex was seen. In scan 8 the previously detected RSN in right posterior parietal and temporal cortex extended into more inferior brain regions.

### Cardiac-related pulsatility

**Figure 12:**
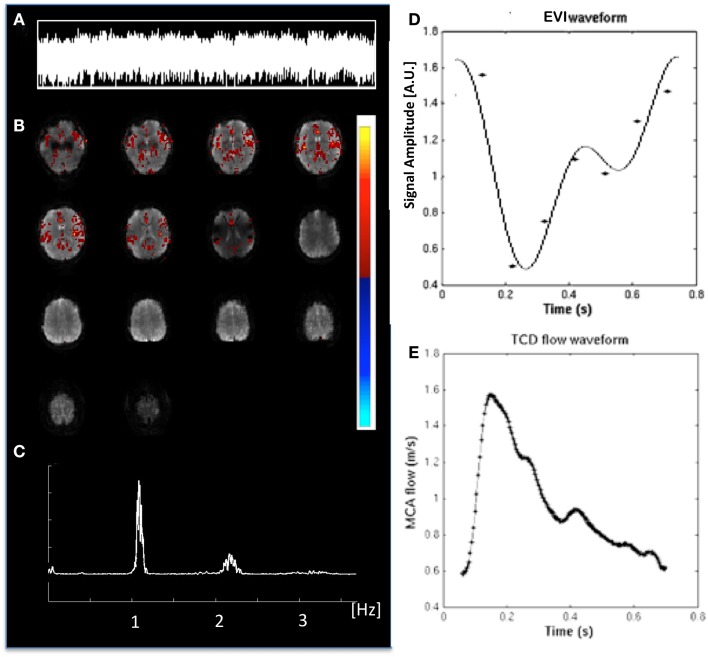
**Cardiac-related signal pulsation measured in a healthy control using MEVI2 (TR: 136 ms)**. **(A)** ICA time course of the pulsation. **(B)** ICA spatial map shows pulsation predominantly in insular cortex and medial gray matter (*z*_max_ = 12.5). The color scale for ICA is shown on the right. **(C)** Corresponding raw power spectrum shows the cardiac frequency and its first harmonic. **(D)** Fitted MEVI2 signal time course, which is inverted with respect to **(E)** a typical Transcranial Doppler Ultrasound waveform from the middle cerebral artery (from Wagshul et al., 2011).

**Figure 13:**
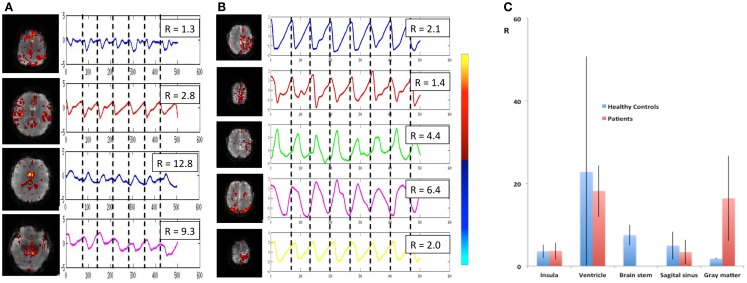
**Cardiac-related signal pulsation in different brain areas measured using MEVI2 (TR: 136 ms) in (A) a healthy control and (B) patient 3 with an AVM**. Selected slices from the spatial ICA show regions with cardiac-related signal pulsation [*z*_max_ ranging from 7.7 to 14.4 in **(A)** and from 5.3 to 14.4 in **(B)]**. Interpolated ICA time courses within a 6.8-s window showing remarkable morphological waveform differences and shifts in phase in the AVM and in adjacent gray matter regions. The ratio *R* of the power spectrum amplitudes at the cardiac frequency versus the first harmonic is shown in the insets. **(C)** Group analysis across healthy controls (*n* = 9) and patients (*n* = 6) shows regional differences in *R* (ratio of the power spectrum amplitudes at the cardiac frequency versus the first harmonic). The color scale for ICA is shown between **(B)** and **(C)**.

Cardiac-related physiological signal fluctuation in healthy controls was mapped into clearly separated ICs in insular cortex, cortex, sagittal sinus, brain stem, and CSF. Several cardiac-related ICs of vascular origin with *Z*-scores ranging from 8.1 to 20.7 were detected in insular cortex, cortex, sagittal sinus (Figure 12), in addition to pulsation in the brain stem (*Z* = 22.2) and in the ventricles (*Z* = 16.5). This pulsation, which was detected on a beat-by-beat basis, was synchronous with peripheral pulse recording throughout the entire scan. Power spectra showed significant amplitude at the first harmonic and in some cases also at the second harmonic (Figure 12C). The waveform of the cardiac-related signal pulsation in insular cortex (Figures 12D,E) was inverted with respect to typical Transcranial Doppler Ultrasound (TDU) and phase contrast MRI waveforms obtained from the middle cerebral artery (e.g., Wagshul et al., 2011), which suggests that the cardiac-related signal pulsation in our MEVI data is dominated by BOLD contrast rather than in-flow effects as usually assumed for BOLD contrast fMRI (e.g., Kruger and Glover, 2001). The signal pulsation in our data is also consistent with the pulsation waveform measured in cingular cortex in one of the early studies using conventional EPI (Dagli et al., 1999). The first harmonic of the cardiac-related pulsation was stronger in components with vascular origin (insula, sagittal sinus) compared to components originating from the ventricles and the brain stem (Figure 13). Multiple ICs with strongly enhanced cardiac-related signal pulsation were measured in *patient 1* with a brain tumor and in *patient 3* with an arterio-venous malformation (Figure 13B). Distinct time shifts on the order of 100 ms were measured between cardiac-related ICs in and adjacent to the AVM, which reflect different phases of the cardiac-related pulse wave propagation. The statistical analysis showed a trend (*t*-test, *p* = 0.14) toward a larger amplitude ratio *R* in gray matter in patients compared with healthy controls (Figure 13C).

## Discussion

### ICA and seed-based connectivity

Mapping of intrinsic signal variation mostly in the low-frequency band<0.1 Hz has emerged as a powerful tool and adjunct to task-related fMRI and DTI-based fiber tracking for mapping functional connectivity within and between RSNs (Fox et al., 2005; De Luca et al., 2006; Raichle and Snyder, 2007; Schopf et al., 2010; Li et al., 2011). Recent studies have shown that dozens of different RSNs can be measured across groups of subjects (Abou-Elseoud et al., 2010; Allen et al., 2011). However, source separation with ICA in individual subject data using conventional EPI is limited by the contrast-to-noise ratio of the signal fluctuations and aliasing of cardiac- and respiration-related signal fluctuations, which requires model-based retrospective deconvolution methods (Glover et al., 2000). Our data using MEVI and ICA show that a considerable number of RSNs that have been mapped in a recent group study (Allen et al., 2011) can be identified in single subjects. Our data also show that source separation in single subjects exhibits considerable inter-individual variability. This variability may reflect inter-individual differences in dynamic cycling between different FNC states, including hypersynchronization, drowsiness, and low synchronization (Allen et al., 2012), as well as in neurovascular coupling and physiological signal fluctuation. Physiological noise correction might further improve ICA analysis, in particular in data sets that exhibit low contrast-to-noise ratio in the RSN signal time courses. Given the spatial heterogeneity in cardiac-related signal pulsation shown in our study this approach will require a comprehensive analysis of ICA source separation as a function of contrast-to-noise ratio in the RSN, respiration, and cardiac frequency bands using regionally adaptive signal pulsation models. This approach will be explored in a future study. Movement during the fMRI acquisition is a major confound for resting-state connectivity studies obscuring networks as well as creating false-positive connections (Satterthwaite et al., 2012; Van Dijk et al., 2012) despite state-of-the-art motion “correction” in post-processing. Monitoring these dynamics in real-time to assess data quality is expected to improve consistency of data quality in clinical research studies and our understanding of the underlying neurophysiological mechanisms.

Seed-based correlation analysis (Van Dijk et al., 2010) and spatial ICA (Calhoun et al., 2001) are the principal tools to map functional connectivity, which have been shown to provide similar results (Van Dijk et al., 2010; Erhardt et al., 2011). Seed-based connectivity measures have been shown to be the sum of ICA-derived within- and between-network connectivities (Joel et al., 2011). Seed-based correlation analysis is suitable for real-time resting-state fMRI due to the high sensitivity of correlation analysis and straightforward interpretation of results (Cole et al., 2010a). In contrast, data driven approaches, such as ICA, in single subjects may require considerable user interaction to interpret resulting maps and time courses. Semi-automated data sorting routines for ICA are under development, but actual real-time applications have not yet been demonstrated (Soldati et al., 2013a,b). A model-based approach such as seed-based correlation analysis that uses prior knowledge is advantageous compared to ICA for detecting small signal changes. However, seed-based techniques are sensitive to the choice of the seed regions (Cole et al., 2010a). Furthermore, seed-based correlation analysis requires regression of confounding signals, which typically include the six parameters of motion correction and their derivatives, and the average signal from up to three brain regions (whole brain over a fixed region in atlas space, ventricles, and white matter in the centrum semiovale). Regression of these signals is computationally intensive and may remove RSN signal changes that are temporally correlated with confounding signals. Here we introduce the combination of seed-based sliding-window correlation analysis with a meta-statistics approach that employs a running mean and standard deviation (Welford, 1962) across dynamically updated correlation maps to generate cumulative meta-statistics maps. Our data show that this meta-statistics approach provides strong rejection of confounding signals from head movement, respiration, cardiac pulsation, and signal drifts (Figure 3) and high sensitivity for mapping inter- and intra-network connectivity dynamics at time scales as short as 1 s without the need for regression of confounding signals (Figures 6 and 7). Furthermore, this methodology is suitable for real-time mapping of FNC dynamics as shown in Figures 5 and 8.

Independent component analysis on the other hand is a powerful data driven approach that has been applied in many group studies and is suitable for single subject analysis (Koopmans et al., 2012). ICA also performs spatial filtering, which enables segregation of spatially overlapping components. However, source separation with ICA is sensitive to the selection of the model order, which is *a priori* unknown and necessitates dimensionality estimation approaches, such as the MDL, BIC, and AIC (Calhoun et al., 2001; Li et al., 2007). Furthermore, automated ordering of ICA components to enable consistent identification of RSNs is challenging. Using the MDL criterion to determine the model order resulted in a relatively small number of ICs relative to the large number of time points in a MEVI scan. At shorter simulated scans times the ICA was less able to separate sources and we found that multiple RSNs were merged in single ICs. Our resting-state data also suggest that using a larger number of components than provided by the MDL criterion may be advantageous for separating RSNs that are co-localized in a single IC in some of our data. Interestingly, the number ICs detected by the MDL criterion increased considerably when spatially interpolating the data, which suggests that spatial dimensionality independent of spatial information content plays an important role in source separation with ICA. This dependence of ICA source separation on preprocessing warrants further investigation. The effects of increasing model order on the noise level and segregation of RSNs in individual subject data need to be addressed in a future study. Furthermore, it will be of interest to investigate the loss of MEVI information in the initial PCA-based data reduction step. The performance of ICA source separation with high-speed fMRI requires further investigation as sensitivity for detecting and for separating RSNs varied across subjects. For example, in some subjects the ICA time course displayed dynamic mixing and unmixing of different signal sources throughout the entire ICA time course. In other cases a separation of a steady signal pulsation time course into two complementary ICs with time courses that displayed decreasing and increasing pulsation amplitude was observed. Several studies have shown that optimization of the data analysis methodology, such as using back-projection methods, reduces inter-session variability (Smith et al., 2005; Chen et al., 2008). Further work across larger groups of subjects is thus necessary to assess the reproducibility of source separation in single subjects.

There is now increasing evidence that RSNs are not stationary (Hou et al., 2006; Kang et al., 2011), which has attracted considerable interest in recent studies (Chang and Glover, 2010; Scholvinck et al., 2010; Allen et al., 2012). However, the neural correlates of resting-state fluctuations in fMRI are not well understood and are a focus of current research (Morcom and Fletcher, 2007; Shmueli et al., 2007; Pizoli et al., 2011; Wong et al., 2011). The seed-based real-time sliding-window correlation analysis with meta-statistics developed in this study enables sensitive analysis of fluctuations in resting-state connectivity at much shorter time scales compared to ICA and hypothesis-driven analysis of connectivity between specific nodes of RSNs. The decreases in connectivity fluctuation with increasing sliding-window width measured in our data highlights the advantage of ultra-high-speed fMRI for characterizing the temporal dynamics of resting-state connectivity and for monitoring transitions between resting states. It also emphasizes that averaging across several minutes of a resting-state scan may underestimate the maximum strength of functional connectivity between regions that exhibit strongly fluctuating connectivity.

Our seed-based sliding-window correlation analysis combined with meta-statistics revealed considerable short-term temporal fluctuation of intra- and inter-network FNC between positive and negative values at short time scales. FNC averaged across an entire 5 min scan was predominantly positive across subjects as shown in Figure 8, which is consistent with previous studies demonstrating positive overall correlation between RSN time courses before regression of the global mean (Fox et al., 2009; Murphy et al., 2009). A recent study demonstrated that correlation coefficients between the PCC and its anti-correlated regions without global regression were substantially weaker than those of the positive correlations within regions of the DMN, consistent with previous studies (Chang and Glover, 2010). That study also showed considerable fluctuation in signal correlation at time scales of 2 and 4 min. The observed anti-correlation between the DMN and task-positive networks remains a topic of ongoing investigation and intense debate with regards to the validity of global signal regression (Fox et al., 2009; Murphy et al., 2009; Uddin et al., 2009; Cole et al., 2010b; Chai et al., 2012). With seed-based connectivity the observation of correlations and anti-correlations is highly dependent on the choice of seed locations. Future studies will have to more thoroughly investigate correlations with a wider range of seed locations. In summary, the measurement of short-term correlations and anti-correlations at time scales much shorter than those reported in previous studies, using high-speed fMRI without global signal regression, will facilitate the characterization of the neurobiological basis of the observed anti-correlations.

Monitoring RSN fluctuations online in correlation with other observables of subject behavior and state would provide a new approach for studying the physiological and cognitive correlates of resting-state fluctuations. Our real-time methodology enables experimental neurofeedback based on intra- and inter-network connectivity, which may provide a means for self-controlling the temporal dynamics of resting-state fluctuations. For example, by controlling activation of task-positive networks it may be possible to modulate the anti-correlated default mode RSN, which may have implications for cognitive behavioral therapy.

### Sensitivity of MEVI

Inline with recent studies, we show that the detection of major RSNs and separation of physiological signal fluctuation in single subjects is facilitated by the high temporal resolution MEVI, which avoids aliasing of cardiac- and respiration-related signal fluctuations, and by the high BOLD sensitivity of MEVI (Posse et al., 2012). As recent studies have shown high temporal resolution improves separation of RSNs using ICA (Smith et al., 2012) and may facilitate detecting the temporal dynamics of RSNs at frequencies above 0.1 Hz (Boubela et al., 2013; Boyacioglu et al., 2013; Chu et al., 2013; Lee et al., 2013), which as a recent study suggests may exhibit greater spatial and temporal stability than low-frequency connectivity (Lee et al., 2013). Consistent with these studies our data show that MEVI improves separation of RSNs and facilitates detecting the higher frequency ranges of resting-state signal fluctuation, which as our data show extend up to 0.27 Hz.

High-speed fMRI reveals respiration-related signal changes at the edges of the MEVI slabs, which may be due to movement, B_0_-shifts, or a combination of both, whereas the center of the slabs was free of these signal changes. This spatial separation of respiration-related artifacts represents a distinct advantage of 3D encoding with MEVI compared to multi-slice EPI, where these signal changes are not spatially separable and may thus be more difficult to remove.

### Clinical feasibility studies

There is now increasing evidence that alterations in functional connectivity are detectable in neurologic (Bettus et al., 2010; Pereira et al., 2010; Luo et al., 2011; Negishi et al., 2011) and psychiatric (Greicius, 2008; Broyd et al., 2009) disorders, which may have diagnostic value. The clinical cases in this study demonstrate that high-speed fMRI has high sensitivity for mapping major RSNs and disease-related changes in functional connectivity in individual patients. Spatial displacement of major RSNs and reduced connectivity within RSNs was mapped in the vicinity of brain tumors and vascular malformations. Resting-state fMRI is particularly advantageous for mapping the sensorimotor cortex in patients with motor impairment, which may be challenging with task-based fMRI due to attention-related unspecific activation and dysregulation of cerebro-vascular coupling in the vicinity of brain lesions. Localization of sensorimotor cortex in patients with motor disability and in the vicinity of brain lesions with impaired cerebro-vascular coupling was more focal in resting-state fMRI compared with task-based fMRI. Segregation of the sensorimotor RSN into laterality-specific subnetworks in patients with brain tumors and AVMs in this study suggests disruption of functional connectivity in the sensorimotor cortex. This dynamic measure of functional integration is complementary to the static connectivity metric obtained with fiber tracking in DTI. Our data show that disease-related changes in resting-state connectivity in the vicinity of brain lesions may manifest as decreases or increases in connectivity between nodes of major RSNs, or even as separate lesion-specific RSNs. Anti-correlations between the DMN and task-positive networks that may be affected by brain lesions provide insights into competitive mechanisms that control resting-state fluctuations (Fox et al., 2005; Uddin et al., 2009). Inter-individual variability in connectivity may be elevated by certain disease conditions, in particular in the vicinity of brain lesions known to impair neurovascular coupling and in brain regions with inflammation. For example, gliomas may be associated with mass effect that can distort anatomy, and may affect eloquent cortex function by tumor infiltration and abnormal neurovascular coupling, generally greater with higher grade, potentially compromising detection of BOLD fMRI signal (Holodny et al., 2000; Hou et al., 2006; Jiang et al., 2010). While resting-state fMRI may provide a sensitive approach for studying neurovascular correlates of disease processes that is complementary to structural MRI and DTI, further studies are required to characterize the specificity of this connectivity information and to quantitatively assess the impact of altered cerebro-vascular reactivity in the vicinity of brain lesions on resting-state connectivity. As a range of pathological tissue changes, such as hyperplasia, inflammation, and edema, may impact apparent resting-state connectivity, it is necessary to investigate whether these changes are indeed indicative of true changes in functional connectivity or whether they are a side-effect of changes in regional cerebro-vascular reactivity.

In two of our patients with epilepsy it was feasible to monitor dynamic changes in major RSNs and the emergence of a separate RSN associated with cortical dysplasia. In *patient 7* with cortical epilepsy a separate RSN emerged dynamically in right posterior parietal and temporal cortex, a region that exhibited interictal spike activity. While these findings may be related to interictal spike activity during the scans, a more definitive assessment requires concurrent EEG-fMRI, which is under development in our laboratory. The high sensitivity of high-speed fMRI is expected to be advantageous for studying the infrequent hemodynamic responses to interictal spike activity in patients with epilepsy compared to conventional EPI. MEVI is compatible with the standard 12-channel head array coil that accommodates an EEG cap. It employs small flip angle excitation resulting in low RF power levels, which minimizes saturation of the EEG amplifiers.

### Cardiac-related pulsatility

Only recently was arterial pulse wave propagation mapped with fMRI (Tong and Frederick, 2012). There is increasing evidence that aging, hypertension, dementia, and Alzheimer disease may have a common microvascular origin and that traumatic brain injury is associated with microvascular damage (Wagshul et al., 2011). However, lack of a non-invasive method capable of assessing pulsatile blood volume in small resistance arteries proves to be the limitation to investigate cerebral microvessels (Wszedybyl-Winklewska et al., 2011).

Our data show that cardiac-related signal pulsation has region specific waveforms and may carry clinically relevant functional information about cerebro-vascular pulsatility in cortex and in vascularized brain lesions. The high temporal resolution of MEVI enables measurement of the pulsation waveform on a beat-by-beat basis using spatial ICA. Increasing the temporal resolution of MEVI to 50 ms is desirable to more fully resolve regional differences in the pulsation waveform and in the phase of the pulse wave propagation. This real-time approach is complementary to phase contrast MRI and TDU as it extends the measurement of cardiac-related pulsatility into gray matter and enables monitoring of dynamic changes in pulsatility waveform.

## Conclusion

We have shown that ultra-high-speed resting-state fMRI is a sensitive tool for presurgical mapping of connectivity within the sensorimotor network, which is complementary to task-based fMRI. Preliminary results in patients with neurological disease demonstrate high sensitivity for monitoring altered resting-state connectivity in the vicinity of brain lesion. Localization of sensorimotor cortex in patients with motor disability and in the vicinity of brain lesions with impaired cerebro-vascular coupling is more focal in resting-state fMRI compared with task-based fMRI, which is advantageous for presurgical mapping. Resting-state fMRI thus provides unique insights into altered functional connectivity associated with brain lesions, which is advantageous for presurgical mapping. Ultra-high-speed fMRI also enables whole brain online monitoring of vascular pulsation and may be useful to assess alterations in arterial pulse wave propagation and vascular compliance in patients with neurological diseases.

The multi-slab EVI pulse sequence and the TurboFIRE software tool are available for research use. Please contact the corresponding author for additional information.

## Conflict of Interest Statement

The authors declare that the research was conducted in the absence of any commercial or financial relationships that could be construed as a potential conflict of interest.

## Footnotes

^1^http://mialab.mrn.org/software/gift/

^2^http://www.fil.ion.ucl.ac.uk/spm/

^3^http://www.cis.hut.fi/projects/ica/icasso/

^4^http://www-sop.inria.fr/asclepios/software/MedINRIA/
